# Vitamin D, the immune system, and its relationship with diseases

**DOI:** 10.1186/s43054-022-00135-w

**Published:** 2022-10-17

**Authors:** Nevin Sanlier, Merve Guney-Coskun

**Affiliations:** 1 Nutrition and Dietetics Department, Faculty of Health Science, Ankara Medipol University, Ankara, 06050 Turkey; 2 Nutrition and Dietetics Department, Faculty of Health Science, Istanbul Medipol University, Istanbul, Turkey

**Keywords:** Diseases, Health, Immune system, Vitamin D

## Abstract

**Background:**

Vitamin D is classified as an immunomodulatory hormone that is synthesized because of skin exposure to sunlight. It is known to come into play during the regulation of hormone secretion, immune functions, cell proliferation, and differentiation. Its deficiency can cause many diseases and their associated pleiotropic effects. In addition, in relation to its eminent function as regards adaptive immune response and innate immune response, vitamin D level is associated with immune tolerance.

**Methods:**

Literature search prior to May 2021 was conducted through selected websites, including the MEDLINE, Embase, Web of Science, Cochrane Central, www.ClinicalTrials.gov, PubMed, Science Direct, Google Scholar, and EFSA.

**Results:**

Vitamin D is found effective for the regulation of hormone secretion, immune functions, and cell proliferation along with differentiation. Its role as an immune modulator is based on the presence of receptors on many immune cells and the synthesis of its active metabolite from these cells. Vitamin D, an immune system modulator, inhibits cell proliferation and stimulates cell differentiation. A fair number of immune system diseases, encompassing autoimmune disorders alongside infectious diseases, can occur because of low serum vitamin D levels. Supplementation of vitamin D has positive effects in lessening the severity nature of disease activity; there exists no consensus on the dose to be used.

**Conclusion:**

It is figured out that a higher number of randomized controlled trials are essential to evaluate efficacy pertaining to clinical cases, treatment duration, type, and dose of supplementation and pathophysiology of diseases, immune system functioning, and the effect of vitamin D to be administered.

## Background

Vitamin D is the only vitamin that acts as a hormone and is synthesized in the skin through sunlight exposure. The daily requirement of vitamin D can only meet 10–20% by dietary intake. Vitamin D deficiency has pleiotropic effects on the human body and is associated with related health and diseases such as bone and dental health, cardiovascular disease, hypertension, some types of cancer, types 1 and 2 diabetes, obesity, multiple sclerosis, cognitive decline, dementia, depression, rheumatoid arthritis, allergy, frailty, infectious diseases, and autism. The association with these diseases suggests that vitamin D is a fundamental immune modulator [1, 2]. Vitamin D turns out basically effective during the regulation of hormone secretion, immune functions, cell proliferation, and differentiation. Its immunomodulatory role is based on the presence of receptors on many immune cells and the synthesis of its active metabolite from immune cells [2]. In this context, this review was prepared so as to review the possible influence of vitamin D over the immune system and its relationship with the existing literature.

## Introduction

The vitamin D receptor (VDR) together with metabolizing enzymes is expressed by a variety of immune cells: lymphocytes, monocytes, macrophages, and dendritic cells to name a few (DC) [3]. It responds to innate and adaptive immunity by providing immunomodulation of monocytes, macrophages, dendritic cells (DC), and T and B lymphocytes. 1,25(OH)_2_D acts as a strategic agent in the regulation of immune system homeostasis [4].

Although vitamin D suppresses the activation of the adaptive immune system, it activates the innate immune system, especially monocytes and macrophages. Vitamin D, an immune system modulator, inhibits cell proliferation and stimulates cell differentiation. Thus, while innate supports antimicrobial functions in the immune system, it reduces inflammatory activity and the capacity to initiate an adaptive immune response [5–8]. The major impacts belonging to vitamin D regarding the regulation of immune functions are known as the presence of VDR in active inflammatory cells as well as the ability of the active form of vitamin D to inhibit T-cell proliferation [2, 9, 10]. Vitamin D increases anti-inflammatory cytokine levels (IL-4, IL-5, IL-10, TGF beta) via stimulating T-helper (Th) 2 cells on the inflammatory system and by inhibiting Th1 and Th17 cells and proinflammatory cytokines (IL-2, IL-3, IFN-gamma, TNF alpha) production. Addedly, 1,25(OH)_2_D has an inhibitory effect on the maturation of dendritic cells by preventing the dissimilitude of B-cell precursors into plasma cells. CD4 T cells can transform into regulatory (Treg) and suppressor T cells as well as Th1 and Th2 cells. Active vitamin D increases the conversion of CD4 T cells into Treg cells. In cases where there exists no vitamin D, the number and activity of Treg decrease, and the incidence of autoimmune diseases increases [9–12]. It is stated to have anti-infective properties against infectious diseases, and there is an interaction between the host immune response against pathogens and vitamin D signaling^5^. In response to bacterial infection, VDR and 1-alpha hydroxylase activity increase in monocytes and macrophages. Thus, a serious resistance is formed against intracellular microorganisms such as mycobacteria [6, 7]. It has been reported that by providing immunomodulation of monocytes, macrophages, and DCS Apache with T and B lymphocytes, it responds to innate and adaptive immunity [4]. A healthy immune system is the most important weapon against viral infections [8]. However, when there is an irregularity in the response of immune system toward viral mechanisms, the rate of the inflammatory process increases, which leads to death. It has been shown that it can produce an irregular immune response, especially on lymphocytes [9]. Vitamin D is believed to enhance natural immunity by increasing antimicrobial peptides, namely cathelicidin as a response to infection [10]. Relatively, recent research reveals that vitamin D is indeed a prominent element of the immune system since it actively takes part in the blocking of infections of several sorts also regulating the operations within the immune system [11, 12]. Vitamin D is in fact necessary for monocyte function. The very role of vitamin D during immune system regulation has become more established through the following discovery: VDRs are found in virtually all immune system cells, including antigen-presenting cells like activated CD4 + and CD8 + T cells, B cells, neutrophils, monocytes, macrophages, and DCs. Many immune system cells, such as monocytes, macrophages, DC, and B and T lymphocytes, have the capacity to express CYP27B1 and translate 25(OH)D to 1,25(OH)_2_D. Vitamin D is crucial for the production of AMPs locally through the 1,25(OH)_2_D/VDR complex against infection [4, 13]. The linkage of monocytes and vitamin D is realized by CYP27B1 enzyme [14]. Monocytes perform immune reactions through phagocytizing foreign body in conjunction with the use of toll-like receptors (TLRs) as well as pattern recognition receptors pertaining to some other classes with a view to recognizing foreign presence. It has been proved that there is elevation of CYP27B1 activity when this event occurs. The very situation indicates an increase in 1,25(OH)_2_D, which is locally produced, which binds up to the endogenous VDR and monitors gene expression in monocytes [15]. VDR-driven innate immunity is mediated by CYP27B1 through stimulation of TLR1/2 pathogen-recognizing receptors (PRR) and activation of TLR-4 ligand lipopolysaccharides (LPS) [4, 13, 16]. Pathogens are identified by pathogen-associated molecular patterns (PAMPs) that bind to these PRRs. Vitamin D participates in the differentiation of monocytes into macrophages as well as natural immunity by increasing the phagocytic capacity of macrophages. It creates a natural immune response by fighting pathogens with TLR2 and TLR4 inhibition [4, 5, 13, 16].

**Fig. 1:**
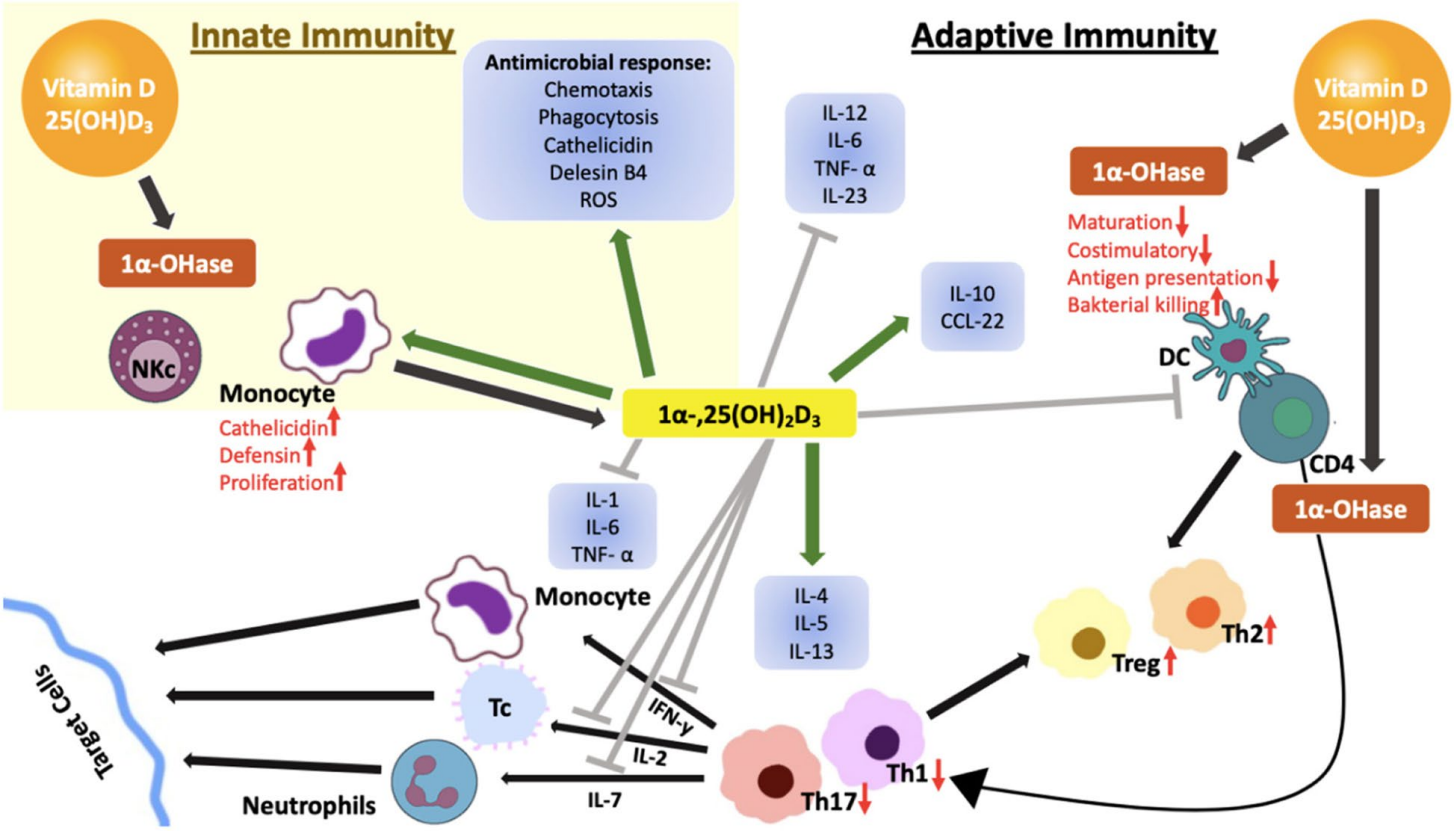
Effects of 1,25(OH)_2_D on innate and adaptive immunity

In light of the aforementioned situations, the relevant information pinpoints the importance of vitamin D whilst protecting the organism against pathogens [17]. Considering the highly significant function of vitamin D for both adaptive immune response and innate immune response, vitamin D level is associated with immune tolerance [18, 19]. These effects on immune cells may explain the beneficial effect of vitamin D observed against some autoimmune diseases [10]. However, although vitamin D supplementation has beneficial effects on reducing the severity of disease activity, there is no consensus on the dose to be used. More randomized controlled trials are needed to evaluate clinical efficacy, duration of treatment, type, and dose of the compound to be administered. Impacts of 1,25(OH)_2_D on innate in tandem with adaptive immunity are illustrated in Fig. 1 [20, 21].

## Methods

Literature search prior to May 2021 was conducted through selected websites, including the MEDLINE, Embase, Web of Science, Cochrane Central, www.ClinicalTrials.gov, PubMed, Science Direct, Google Scholar, EFSA, plus the World Health Organization (WHO). Reference articles were searched and reviewed by the following keywords: vitamin D absorption, metabolism, health effects/benefits, effects/health benefits, vitamin D deficiency, VDR, and 25-hydroxyvitamin D, insulin, and diabetes mellitus obesity, immunity, infection, immune system, autoimmune diseases, autoimmune thyroid diseases, multiple sclerosis, inflammatory bowel diseases, autoimmune rheumatic diseases, rheumatoid arthritis, psoriasis, infectious diseases, COVID-19 and vitamin D, and vitamin D replacement therapy. The same keywords are searched by combinations, disease names, using gene, polymorphism, and other immune disease names. Sub-references of the articles selected according to the keywords were searched, and these articles were also reviewed. Animal studies and clinical human studies on vitamin D treatments and immune diseases were primarily reviewed. These research articles, reviews, reviews of systematic nature, and meta-analyses constituted the groundwork of the current research.

## Result and discussion

### Vitamin D and disease interaction

**Fig. 2:**
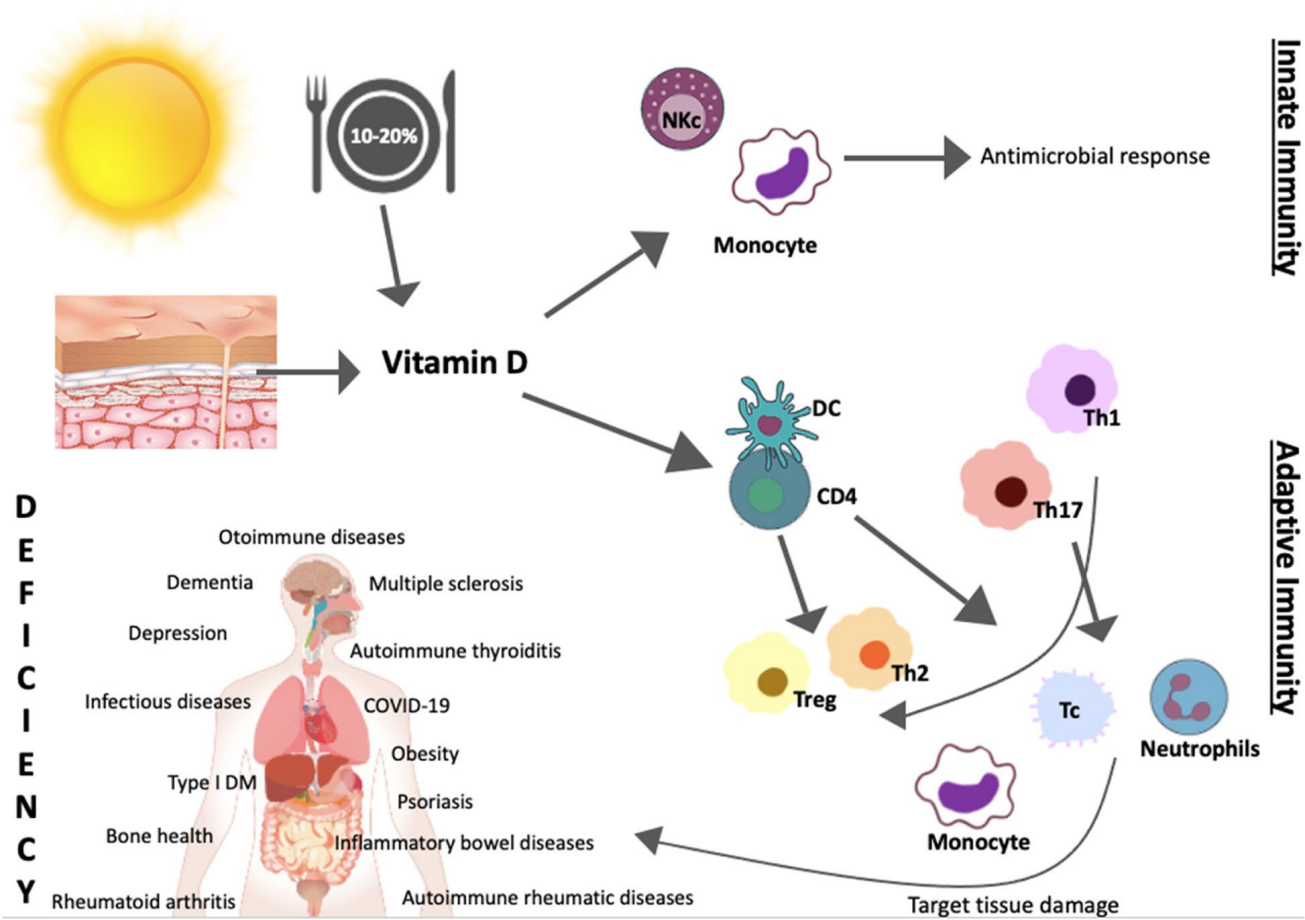
Vitamin D and disease relevance

The relation between vitamin D and disease is shown in Fig. 2.

### Obesity and vitamin D

Vitamin D functions during extracellular Ca concentration regulation and calcium entry into pancreatic β cells. With this mechanism, the synthesis of vitamin D receptor besides 1-alpha-hydroxylase enzymes is stimulated in β cells that secrete insulin from the pancreas, while insulin production and secretion are also stimulated. Further to that since the renin-angiotensin system activity increases in cases of increased adiposity, accordingly, low adiponectin levels are detected in the adipose tissue, and an increase can be achieved through vitamin D impact. High circulating levels of leptin and interleukin-6 can inhibit vitamin D synthesis [22]. A bidirectional association is corroborated for vitamin D and obesity. It is thought that adipose tissue is metabolically active tissue and is able to coordinate vitamin D, or that vitamin D can execute the aforementioned act for adipose tissue [23]. Verily, vitamin D directly suppresses the expression of peroxisome proliferator-activating receptor γ2 (PPARγ2), which provides lipogenesis and differentiation of preadipocytes. It has been reported that 1,25(OH)_2_D suppresses lipogenesis and blocks preadipocyte differentiation by stimulating insulin-dependent gene-2 (In-sig-2) expression. With these key factors, 1,25(OH)_2_D can control adipose storage [24]. Vitamin D can affect obesity through cell signaling mechanism [25]. A consistent relationship was found between increasing body mass index and decreasing serum 25(OH)D concentration [26]. Vitamin D, in its active form, 1,25(OH)_2_D, supports the synthesis of catecholamines by activating the gene expression of the tyrosine hydroxylase enzyme. It has also been suggested that it may contribute to cholinergic functions by increasing the choline acetyltransferase enzyme activity, which is the key enzyme in acetylcholine synthesis [27]. It has been suggested that high adiposity is associated with low serum vitamin D, and weight loss reduces peripheral uptake and leads to an increase in vitamin D concentration [28]. Low vitamin D level increases the differentiation of preadipocytes into mature adipocytes. Mature adipocytes cannot express VDR. The vitamin D receptors are expressed in adipocytes and aside from this are sensitive toward the fat-soluble 1,25(OH)_2_D vitamin. Because vitamin D metabolites are fat soluble, they are retained in adipose tissue. Vitamin 25(OH)D and its conversion to inactive metabolites cause exaggerated hypovitaminosis in obese individuals [29].

### Type 1 diabetes mellitus and vitamin D

Relevant research has underpinned that vitamin D is effective in increasing insulin secretion and insulin sensitivity, and a negative relationship between low serum 25(OH)D levels and prediabetes, diabetes, and metabolic syndrome is evident. As known, vitamin D can affect insulin resistance and β-cell function directly through the VDR and indirectly by affecting calcium homeostasis. It is stated that the presence of vitamin D-dependent calcium-binding protein in pancreatic tissue coupled with the relationship between vitamin D innate and adaptive immunity is influential in this mechanism of action [30]. The influence of vitamin D over glucose metabolism includes changes in extracellular and intracellular calcium concentrations in pancreatic β cells. Insulin secretion is considered as a calcium-dependent process that is mediated by 1,25(OH)_2_D with the addition of parathyroid hormone (PTH). It has also been punctuated that serum 25(OH)D deficiency and consequent increase in PTH may cause difficulties in the capacity of β cells to convert pro-insulin to insulin, and diabetes may develop with apoptosis of cells [31]. The deficiency of vitamin D has been ascribed to boosted insulin resistance, to reduced insulin production, and also to metabolic syndrome^1^. It has been reported that calcitriol supplementation decreases antibodies’ serum levels and delays β-cell destruction succession. Therefore, complement through vitamin D or analogues of it becomes preventive instead of being curative against the disease [32]. A systematic meta-analysis reported improvements in glycemic control indices after vitamin D supplementation in diabetic patients and supported its use as adjunctive therapy against this disease [33]. Amongst the environmental factors considered protective against type 1 DM is the early intake of vitamin D supplementation. One other meta-analysis demonstrated 1 DM risk was lessened in infants who were given vitamin D supplements, to a considerable extent as opposed to those who were not. The dose–response impact is also verified, as those who use relatively bigger amounts of vitamin D have a diminished risk of developing type 1 DM [34]. That being said, studies evaluating the gains out of maternal vitamin D supplementation in preventing type 1 diabetes in children could not show this relationship [35]. In conclusion, intensifying intake of vitamin D intake during early childhood years to assure serum 25(OH)D stay within the optimal range appears to be a guard against developing type 1 diabetes. Supplementation of vitamin D assists in controlling disease activity, if not curative. With that being said, no affirmation is pertinent to the longer-term impacts of supplementation of vitamin D over morbidity or mortality speaking of patients with type 1 diabetes [36]. In recent years, genes mixed up in vitamin D metabolism have also attracted attention due to their relationship with vitamin D deficiency. For example, susceptibility to type 2 diabetes was reported to increase with the VDR gene. Additionally, vitamin D deficiency is found to be linked to microvascular complications of diabetes such as neuropathy and retinopathy [37].

### Sjogren’s syndrome and vitamin D

Sjögren’s syndrome is an autoimmune disease which usually impacts the exocrine glands and glandular manifestations in around half of the related patients. Vitamin D levels, in lowered forms, were found in patients with Sjögren’s syndrome in contrast to control groups [38]. However, another study found no significant difference [39]. Although there is no consensus over vitamin D supplementation’s impact on the patients in question, supplementation is recommended as a prophylactic measure [40].

### Autoimmune thyroiditis and vitamin D

It has been determined that there is an interdependence between deficiency of vitamin D and thyroid autoimmunity [41]. Thyroid hormone is effective in sustaining sufficient vitamin D levels, and the immunomodulatory function of vitamin D may affect developing of autoimmune thyroid disease. Therefore, it was associated with a decline in the levels of anti-thyroperoxidase antibodies, which may have a beneficial impact over these diseases, yet no meaningful correspondence was spotted with others in this sense [42]. In Hashimoto’s thyroiditis, cellular immunity is impaired as a result of genetic defects in suppressor T cells. As a result of this defect, the appropriate T lymphocytes cannot suppress the other T lymphocytes, viz., the helper. Helper T lymphocytes, when activated, bond together B lymphocytes and secrete many cytokines (INF-γ). Activated B lymphocytes construct antibodies that respond with thyroid antigens [43]. Low serum 25(OH)D level is detected in persons with Hashimoto’s thyroiditis. Low serum 25(OH)D level is attributed to high anti-thyroid antibody level, thyroid functions with abnormal characteristics, escalated thyroid volume, and raised TSH level. Nevertheless, a weak-to-none relationship between low vitamin D levels and thyroid autoimmunity has been recorded [44]. In a study, low levels of serum 25(OH)D levels were correlated negatively with serum anti-thyroid peroxidase antibodies in euthyroid patients having Hashimoto’s thyroiditis. Following a 4-month supplementation of vitamin D, serum anti-TPO levels in patients with 25(OH)D levels of 30 ng/mL curtailed in considerable fashion [45].

### Multiple sclerosis and vitamin D

Multiple sclerosis (MS) is categorized as an autoimmune central nervous system (CNS) disease in the literature which is detailed through inflammation, demyelination, with axon damage. MS is a chronic disease. While some of them watch with attacks, some of them watch progressively. Vitamin D in low levels has been allotted to a higher risk of disease development and reoccurrence. Positive effects have been unearthed for vitamin D supplementation as long as doses ranged between 500 and 2000 IU/day and, on top of that, a fall in optic neuritis as well as in the rate of recurrence. Nonetheless, higher doses, that is, 5000–10,000 IU/day, have been denounced for worse outcomes [46]. Supplementation of vitamin D in combination with interferon β has been found to produce a synergistic beneficial effect [32, 47]. In Munger et al.’s (2006) work, it has been shared that a 41.0% reduction in MS risk for every 20 ng/mL (50 nmol/L) an increasement in serum 25(OH)D levels above 24 ng/mL [48]. It has been shown that females consuming more than 400 IU of vitamin D daily have a 41.0% reduction in the risk of MS development [35]. Thereupon, vitamin D deficiency is believed to be a key to developing dysregulated T-helper cells, CTL, NK cells, and B cells, which cause central nervous system autoinflammation which harms neurons and oligodendrocytes inherent in MS [49].

### Inflammatory bowel disease and vitamin D

Deficiency of vitamin D has been assigned to the start of intestinal diseases portrayed with progressive chronic inflammation of the gastrointestinal tract [50]. In Crohn’s disease, vitamin D has been found out to inhibit Th1 production and Th17 T-helper lymphocyte subpopulations and inflammatory cytokines in the gastrointestinal tract, reduce inflammation, and protect the gut microbiota that is of crucial importance in the functioning of the mucosal immune system [51]. Intestinal homeostasis has been imputed to VDR expression that restricts the production of IL-6 by epithelial cells [52]. Vitamin D supplementation is indeed an influential and safe treatment at doses that are to be evaluated through case by case [53]. VDR polymorphisms have been demonstrated in inflammatory bowel diseases (IBD). The most known variations in IBD are BsmI, FokI, TaqI, and ApaI polymorphisms. However, polymorphism may differ according to races. It is clear through the bulk of literature that patients with IBD are more inclined toward vitamin D deficiency, and that these patients have a greater risk of developing osteomalacia, osteoporosis, and fragility fractures [54] since they are not capable of efficiently forming micelles and chylomicrons that allow for the absorption of vitamin D in their gastrointestinal tract [55]. To this end, patients need to be screened for vitamin D deficiency. It is underlined that they can be treated with greater doses of vitamin D to reach a normal serum 25(OH)D level of at least 30 ng/mL (75 nmol/L) [19]. This points to that the relation between vitamin D status and IBD may be of bidirectional character. It has been highlighted that persons residing in lower latitudes have a consistently low risk of developing IBD compared to the ones in high latitudes [56]. The maximum quartile of estimated serum levels of 25(OH)D has been displayed to be associated with a 46% decreased risk of Crohn’s disease and a 35.0% decreased risk of ulcerative colitis [57]. Another meta-analysis study showed that vitamin D supplementation in IBD patients was connected to a alleviated relapse rate, backing up the therapeutic role of vitamin D as a collateral therapy for IBD [58]. Patients with IBD are not able to absorb vitamin D effectively, and hence, their case entails 2–3 times higher doses of vitamin D supplementation to reach normal serum 25(OH)D levels. Sufficing vitamin D supplementation in IBD is recognized as an adjunctive immunomodulatory agent shown to not only limits the risk of osteoporosis, osteomalacia, and fragility fractures but also improves disease activity [36].

### Autoimmune rheumatic diseases and vitamin D

Vitamin D deficiency is commonly found in patients with autoimmune rheumatic disease involving more than 100 inflammatory, degenerative, and autoimmune diseases and is condemned for joint damage, severe pain, disability, and death [59]. De la Torre Lossa et al. (2020) declared that vitamin D deficiency is more common amongst individuals with rheumatoid arthritis and can trigger its beginning or advancement [60]. Mateen et al. (2017) viewed that these patients were with lower calcidiol and higher inflammatory cytokine levels. Calcidiol is discovered to no longer able to perform its immunomodulatory role at depleted concentrations, and that the heightening of cytokines results in the augmentation of disease severity. Notwithstanding, in spite of an improvement in disease activity, findings hitherto appear not sufficient to expound the immunomodulatory role of vitamin D to the full extent [61].

### Rheumatoid arthritis and vitamin D

An inverse relationship was found between serum 25(OH)D and 1,25(OH)_2_D levels and disease activity, severity, and functional disability in patients with rheumatoid arthritis (RA) [62]. In a prospective cohort study, an inverse correlation was found between the risk to develop RA and vitamin D intake [63]. The most common mutations associated with RA are the FokI, BsmI, TaqI, and ApaI polymorphisms in the VDR gene and rheumatoid arthritis. It is depicted with the production of several cytokines, including TNF-α, IL-6, IL-15, IL-17, and IL-1β. Vitamin D is of utmost importance for the etiopathogenesis of RA. By inhibiting Th1 cells, it can reduce the production of IL-1, IL-6, IL-17, and TNF-α and the release of IL-2 and IFN-γ from CD4 cells. Inhibiting IFN-γ secretion downregulates NF-κB and ultimately decreases IL-12 synthesis. Th1 cells and Th17 cells are both rudimentary in the pathogenesis of experimental arthritis [64].

### Psoriasis and vitamin D

Psoriasis is classified as a chronic inflammatory autoimmune disease distinguished by hyperproliferation of VDR-expressing keratinocytes. Amon et al. (2018) remarked low serum vitamin D levels in patients with psoriasis and found low vitamin D concentrations in 44.0% of them [65]. Topical treatment using the vitamin D analog calcipotriol can adjust the expression of proinflammatory cytokines (e.g., TNF-α, IFN-γ, IL-2, and IL-8) as well as psoriasin with coebnericin, proteins which potentiate reactions of inflammatory nature in psoriasis. Conversely, a rise in IL-10, an anti-inflammatory cytokine inhibiting proinflammatory cytokine synthesis by T lymphocytes and macrophages, is produced [66, 67]. Similarly, in another study, it was ascertained that the calcitriol analog maxacalcitol mitigated psoriasiform inflammation of the skin via giving rise to T-regulatory cells and curbing the production of IL-23 and IL-17, cytokines which are foundational for psoriasis, amongst other diseases [68]. It has been delineated that serum 25(OH)D deficiency is amongst the known independent risk factors for psoriasis. The line of research has confirmed a relatively high prevalence of the deficiency of vitamin D amongst patients with psoriasis in contrast to the general population, regardless of the presence of succeeding adjustment for confounders in a multivariate analysis. Patients with psoriasis emerge as the ones who are less likely to expose themselves to sunlight which is the basic resource for vitamin D. On the flip side, treated vitamin D deficiency may be beneficial [69].

### Infectious diseases and vitamin D

Epidemiological data enable to connect vitamin D deficiency to defective functioning of the immune system with proliferated risk of infection as well as susceptibility to autoimmune disease [70]. Relationships have been disclosed between 25(OH)D deficiency and enlarged risk of infection with mycobacterium tuberculosis in parallel with respiratory tract infections, especially in the case of infection [56]. Another study stressed that vitamin D supplementation was protective against acute respiratory tract infections in a population with 25(OH)D deficiency, particularly those who received daily or weekly supplementation [71]. The mechanism through which vitamin D forms a shield against respiratory infections is derived from in vitro research showing that 1,25(OH)_2_D leads to uplifted cathelicidin expression, regulation of cytokine release, and suppression of the adaptive response via inflating innate immunity [72]. Results of the study pointed out that vitamin D, along with antibiotics in children as well as adults had no additional positive impact for the treatment of acute bacterial pneumonia, but children had low baseline 25(OH)D levels [73]. As vitamin D has a significant influence over macrophages, great efforts have been made to bind vitamin D to tuberculosis. 25(OH)D deficiency has been confirmed to boost the risk of active tuberculosis development [74]. Reasons assumed are that 1,25(OH)_2_D results in the activation of macrophages and increased mycobactericidal activity by induction of cAMP and DEFB4 [75]. Also, adding vitamin D supplementation to antituberculosis therapy has turned out to have a positive effect [76]. Even in chronic obstructive pulmonary disease (COPD), patients with COPD have been attested to be more likely to suffer from 25(OH)D deficiency than compatible healthy smokers, with a degeneration of COPD classification and a further disease-related exacerbation rate [77].

### COVID-19 and vitamin D

Severe acute respiratory syndrome coronavirus-2 (SARS-CoV-2) is an enveloped, single-stranded ribonucleic acid (RNA) virus. Coronavirus disease (COVID-19) as an outcome of SARS-CoV-2 was first spotted in Wuhan, China, in late December 2019 and has rapidly spread around the world then [78]. COVID-19 can result in serious infections, pneumonia, kidney failure, and death [79]. Whereas the function of vitamin D has been asserted as paramount with the investigation of the pathophysiology of COVID-19, a lot is yet to be learned as to the role of it in preventing the infection as well as lethality [80]. Vitamin D supports cellular immunity by reducing the cytokine storm experienced as part of the disease. The emergence of vitamin D in relation to COVID-19 is especially related to its effect on T-cell response. In case of viral or bacterial infection, the immune system responds by releasing anti-inflammatory and proinflammatory cytokines. The cytokine storm taking place with the excessive secretion of these cytokines is related with the severity of COVID-19 and is shown as an important cause of COVID-19 mortality [81]. Vitamin D decreases the Th1 response and increases the Th2 and regulatory Th response. Thus, while the release of proinflammatory cytokines decreases, the release of anti-inflammatory cytokines is increased. It has been reported that vitamin D can prevent cytokine storm and acute respiratory distress syndrome with this regulatory effect on the immune system [82]. As another mechanism, it has been proposed that vitamin D may reduce the severity of COVID-19 via rising up the expression of angiotensin-converting enzyme 2 (ACE-2) while reducing pulmonary vasoconstriction [83].

In a study, a relationship was found between vitamin D status and COVID-19 mortality [84]. Although randomized controlled trials and research with large populations evaluating serum vitamin D levels and the severity of COVID-19 still awaiting to be completed, there is proof to share that vitamin D positively affects the very course of the disease. SARS-CoV-2 initially employs immune-avoidance mechanisms, which in some patients are associated with elevated proinflammatory cytokine release, boosted risk of pneumonia [85] sepsis, and acute respiratory distress syndrome, which often results in death [86]. While many factors come into play when determining the outcome of COVID-19 patients, serum vitamin D levels have been shown to be correlated with disease incidence as well as mortality [87]. Conditions associated with vitamin D deficiency, that is to say, diabetes and hypertension, may be indirectly associated with the severity of COVID-19 [88]. Retrospective, multicenter research put forth that COVID-19 patients with vitamin D deficiency had poorer outcomes overall, while those with higher vitamin D levels had better outcomes [89]. Rhodes et al. (2021) accentuated that there is strong evidence to attribute vitamin D deficiency to severity of COVID-19 infection [90]. Another study noted that African Americans with vitamin D deficiency and those with more severe COVID-19 disease may benefit from supplementation [91]. In a smaller cohort observational study placed in Singapore, 43 COVID-19 patients receiving combined oral doses of vitamin D, Mg, and vitamin B12 showed significant protective effects against clinical worsening, even after adjustments made as regards age, sex, and comorbidities [92]. Low vitamin D levels have also been measured in patients with preexisting disease with severe COVID-19 [93]. In Belgium, a retrospective observational study with 186 positive cases and 2717 negative controls found significantly lower serum vitamin D levels in COVID-19 patients in comparison with control subjects [94]. That said, there is not any sufficient evidence to ensure there exists a relation between serum vitamin D levels and COVID-19 severity and mortality, and randomized controlled and large-scale cohort research is required to test the mentioned hypothesis [80].

### Vitamin D and its relationship with other diseases

In addition to activating the antimicrobial defense system in the body, vitamin D also has some anti-inflammatory activities. In monocytes and macrophages, IL-1β, IL-6, IL-8, and TNF have a pivotal effect on reducing the production of proinflammatory mediators and regulating the host inflammatory response against pathogens. Vitamin D performs its biological activity through VDR, and it has been reported that the induced VDR negatively regulates NF-κB activation and reduces the inflammatory response [95]. In addition, it is known that the seal I polymorphism in vitamin D receptors prevents the formation of anti-inflammatory signals originating from vitamin D and predisposes patients to respiratory syncytial virus-associated bronchiolitis. A similar anti-inflammatory effect has been noted for lung epithelial cells infected with influenza virus [96]. Vitamin D at lower levels has also been associated with lifelong immune system development in humans, respiratory infections, wheezing, transfer of human immunodeficiency virus (HIV) in infants, and bacterial vaginosis in pregnancy [6, 97]. More recently, data have been published with respect to the potential function that vitamin D holds respecting an increased resistance to HIV infection. In particular, HIV-exposed seronegative patients produced more cAMP in the oral mucosa and peripheral blood and had higher CYP24A1 mRNA in the vaginal mucosa. CYP24A1 is indicative of high levels of 1,25(OH)_2_D [98]. Low serum vitamin D has been linked with HIV/AIDS progression and mortality [99]. Vitamin D can modulate the innate immune system, as well as enhance the phagocytic ability on immune cells, and strengthen the physical barrier role that epithelial cells carry, specifically 1,25(OH)_2_D, corneal and intestinal epithelial barrier function.

Data on non-mycobacterial infections in humans are also correlated with urinary and respiratory infections and sepsis. It suggests susceptibility to urinary tract infection in children who have low vitamin D levels owing to decreased cAMP production and defense of β2 [100]. In addition to that, cAMP levels and other antimicrobial peptides in patients with COPD have been connected to a widened risk of acute exacerbations [101]. Commensurating with the abovementioned data treatment with 1,25(OH)_2_D is functional in reducing respiratory infections in asthma patients through increased cAMP expression and modulation of inflammatory cytokines [102]. Results reflecting findings on the role of vitamin D status and vitamin D supplementation in sepsis are accessible in pediatric and adult patients: a clear role for 25(OH)D and cAMP in pediatric patients has not been demonstrated. Whereas in adults, 25 levels are lower (OH)D sepsis and cAMP increases, circulating vitamin D increases, and higher doses decrease inflammatory cytokines such as IL-6 and IL-1β [103]. Patients with autoimmune hepatitis also have a high frequency for vitamin D deficiency. Serum vitamin D level was found to be low in patients with chronic liver disease, and there is a negative correlation between vitamin D level and disease progression. Jointly, it has been identified that vitamin D deficiency contributes to the pathogenesis of the disease in these patients in genetic and non-genomic ways. It has been reported that autoimmune liver diseases are associated with BsmI and TaqI polymorphisms. The liver has an important place in the synthesis steps of vitamin D. Having said that, it is not certain if vitamin D deficiency in chronic liver diseases is a cause or a result in disease-related events [104].

Scholars have recognized that vitamin D deficiency is associated with disease activity in patients with systemic lupus erythematosus (SLE). In these patients, photosensitivity provides less sun exposure, reduces the synthesis of vitamin D from the skin, and chronic use of corticosteroids changes vitamin D metabolism. It supports that deficiency of vitamin D deficiency can be an impetus to production of autoantibody raising the risk of developing autoimmune disease in patients with genetic predisposition. Vitamin D supplementation is recommended for the prevention of SLE-related morbidity. It has been underscored that vitamin D supplementation yields integral output, to illustrate, inhibition of DC activation, suppression of IgG production by B cell, and regulation of CD4 helper T-cell responses [104, 105].

## Conclusion and recommendations

Current day signposts that deficiency of vitamin D is a compelling issue of public health. Related research advocates vitamin D has an invaluable role as an immunomodulator. While innate enhances the capacity of the immune system to fight against pathogens, its effect on the modulation of the adaptive immune system is controversial. There are uncertainties about the role of vitamin D in the pathogenesis of autoimmune diseases. It is unclear whether vitamin D deficiency is a cause or consequence of autoimmune diseases. At the same time, the increase in vitamin D deficiency at epidemic rates and its coincident with the increasing prevalence of autoimmune diseases support this relationship. More comprehensive studies are continuing on the pathophysiology of diseases, immune system functioning, and the effect of vitamin D. The important role of vitamin D in maintaining immune balance should not be overlooked. Low serum vitamin D levels are associated with many immune-related diseases, covering autoimmune disorders and infectious diseases. Studies to date show that sustaining a healthy serum vitamin D level is important for regulating the body’s immune function. EFSA (European Food Safety Authority) reported daily adequate intake (AI) as 10 mcg in 7–11 month babies and 15 mcg in all age groups 1 year and older [106, 107]. Clinical studies are called for to further explain the effects of vitamin D in human beings and to reveal how much these risks can be prevented by treatment in individuals on vitamin D supplementation or with deficiency of it.

## Data Availability

Please contact the corresponding author for data requests.
